# Measurement of the acute metabolic response to hypoxia in rat tumours *in vivo* using magnetic resonance spectroscopy and hyperpolarised pyruvate

**DOI:** 10.1016/j.radonc.2015.03.011

**Published:** 2015-09

**Authors:** Joanne E. Bluff, Steven Reynolds, Stephen Metcalf, Tooba Alizadeh, Samira M. Kazan, Adriana Bucur, Emily G. Wholey, Becky A.S. Bibby, Leigh Williams, Martyn N. Paley, Gillian M. Tozer

**Affiliations:** a Tumour Microcirculation Group, Sheffield Cancer Research Centre, Department of Oncology, University of Sheffield, UK; b Academic Unit of Radiology, Department of Cardiovascular Science, University of Sheffield, UK

**Keywords:** Tumour oxygenation, Magnetic resonance spectroscopy, Dynamic nuclear polarisation, Pyruvate metabolism

## Abstract

**Purpose:**

To estimate the rate constant for pyruvate to lactate conversion in tumours in response to a hypoxic challenge, using hyperpolarised ^13^C_1_-pyruvate and magnetic resonance spectroscopy.

**Methods and materials:**

Hypoxic inspired gas was used to manipulate rat P22 fibrosarcoma oxygen tension (pO_2_), confirmed by luminescence decay of oxygen-sensitive probes. Hyperpolarised ^13^C_1_-pyruvate was injected into the femoral vein of anaesthetised rats and slice-localised ^13^C magnetic resonance (MR) spectra acquired. Spectral integral versus time curves for pyruvate and lactate were fitted to a precursor-product model to estimate the rate constant for tumour conversion of pyruvate to lactate (*k_pl_*). Mean arterial blood pressure (MABP) and oxygen tension (ArtpO_2_) were monitored. Pyruvate and lactate concentrations were measured in freeze-clamped tumours.

**Results:**

MABP, ArtpO_2_ and tumour pO_2_ decreased significantly during hypoxia. *k_pl_* increased significantly (*p* < 0.01) from 0.029 ± 0.002 s^−1^ to 0.049 ± 0.006 s^−1^ (mean ± SEM) when animals breathing air were switched to hypoxic conditions, whereas pyruvate and lactate concentrations were minimally affected by hypoxia. Both ArtpO_2_ and MABP influenced the estimate of *k_pl_*, with a strong negative correlation between *k_pl_* and the product of ArtpO_2_ and MABP under hypoxia.

**Conclusion:**

The rate constant for pyruvate to lactate conversion, *k_pl_*, responds significantly to a rapid reduction in tumour oxygenation.

Tumour hypoxia limits efficacy of radiotherapy and some chemotherapy drugs, as well as stimulating tumour progression [1]. Treatment, in turn, can modify tumour oxygenation, including vascular-targeted approaches specifically designed to induce sufficient hypoxia to promote cancer cell death. Several imaging methods have been tested clinically for monitoring tumour hypoxia, such as the use of radio-labelled nitroimadazoles and other redox-sensitive compounds for positron emission tomography [2,3] and electron paramagnetic resonance (EPR) imaging [4]. Magnetic resonance spectroscopy and/or imaging (MRS/MRSI/MRI) methods include oxygen-enhanced MRI, ^19^F oximetry using perfluorocarbons and ^1^H MRI-based blood oxygen-level dependent (BOLD) imaging [3,5]. Pre-clinical developments have been reviewed by Mason et al. [6]. Dissolution dynamic nuclear polarisation (dDNP) of metabolic substrates combined with MRS/MRSI is established pre-clinically for monitoring *in vivo* metabolism [7] and its first clinical use has now been published [8]. This technique potentially provides a complementary MRS/MRSI-based method for monitoring induced changes in tumour metabolism that are influenced by oxygenation status.

Hyperpolarisation increases ^13^C MR signals of substrates by many orders of magnitude, allowing real-time kinetics of metabolism to be followed in tissue without the interfering background signals experienced in ^1^H MRS. In the glycolytic pathway, hyperpolarised ^13^C_1_-pyruvate (PA) has been the most studied substrate for cancer metabolism because of its high, rapid hyperpolarisation and relatively long T_1_ relaxation time [9,10]. Lactate dehydrogenase (LDH) catalyses pyruvate to lactate production, accompanied by oxidation of nicotinamide-adenine dinucleotide (NADH) to NAD^+^. Altered metabolism is a common feature of cancers and many tumour cells metabolise glucose to lactate under aerobic as well as anaerobic conditions, a phenomenon known as aerobic glycolysis or the Warburg effect [11]. Nevertheless, available data suggest that the majority of tumour energy (ATP) production is via oxidative phosphorylation (OXPHOS) [12] and that there is a switch away from OXPHOS towards increased glucose consumption and lactate production under acute hypoxic conditions (an inverse of the classic Pasteur effect) [13,14]. Therefore, dDNP with PA has the potential for directly monitoring the effect of tumour oxygenation on metabolism. The aim of the current study was to establish how the rate constant for tumour conversion of pyruvate to lactate responds to an acute hypoxic challenge in a rat tumour model.

## Methods and materials

### Animal treatment groups

All experiments were conducted in accordance with the United Kingdom Animals (Scientific Procedures) Act 1986 and following published guidelines [15]. Rat P22 fibrosarcoma fragments [16] were implanted subcutaneously into the rear dorsum of male BDIX rats. A cohort of tumour-bearing rats (*n* = 36) was used to determine local tumour oxygen tensions (pO_2_) via probes incorporating an oxygen-sensitive fluorophore (OxyLite™). These measurements were used to establish a suitable inspired gas mixture for inducing tumour hypoxia in subsequent MRS experiments (see Supplementary Materials). A second group of tumour-bearing rats (*n* = 18) was used for MRS experiments, in which the rate constant for conversion of intravenously administered PA into lactate (*k_pl_*) in tumour tissue was estimated under normoxic and hypoxic conditions. A third group of animals (*n* = 28) was used for *ex vivo* measurement of pyruvate and lactate concentrations in tumour tissue following the same gas challenges as used for MRS.

### Animal preparation for MRS

When tumours reached 18 ± 6 mm mean diameter (mean ± SD), rats (300 ± 27 g; mean ± SD) were anaesthetised with isoflurane and a femoral vein, a femoral artery and a tail vein cannulated. Following surgery, rats were heparinised (1500 Units/kg i.v.) and anaesthesia maintained for imaging by propofol (Rapinovet®, Intervet/Schering-Plough Animal Health, Milton Keynes, UK) infused via the tail vein cannula at a rate of ∼40 mg/kg/h. Rectal temperature was maintained at 37 °C throughout surgery and imaging, using a homoeothermic blanket system (Harvard Apparatus, UK).

### Hyperpolarisation of ^13^C_1_-pyruvate and MR experiments

^13^C_1_-pyruvic acid (CIL, Andover, MA or Sigma Aldrich, Gillingham, UK) was hyperpolarised (denoted PA) using a HyperSense polariser as described previously [17], see Supplementary Materials. After dissolution, PA had a final concentration of ∼150 mM. Rats were located at the centre of a 300 mm bore, 7T magnet with 120 mm, 400 mT/m gradients and an Avance-II spectrometer (Bruker Biospin MRI GmbH, Ettlingen, Germany). Arterial blood pressure was measured in the arterial cannula via a pressure transducer (CWE Inc, Ardmore, PA) for calculation of mean arterial blood pressure (MABP). Blood/tumour pO_2_ was manipulated by supplying the inspired gas as normal air or hypoxia (∼10% O_2_; 4% CO_2_: N_2_ balance) via either tracheal intubation or face-mask at 1 L/min commencing ∼3 min prior to PA infusion and maintained throughout the MRS experiment.

**Fig. 1:**
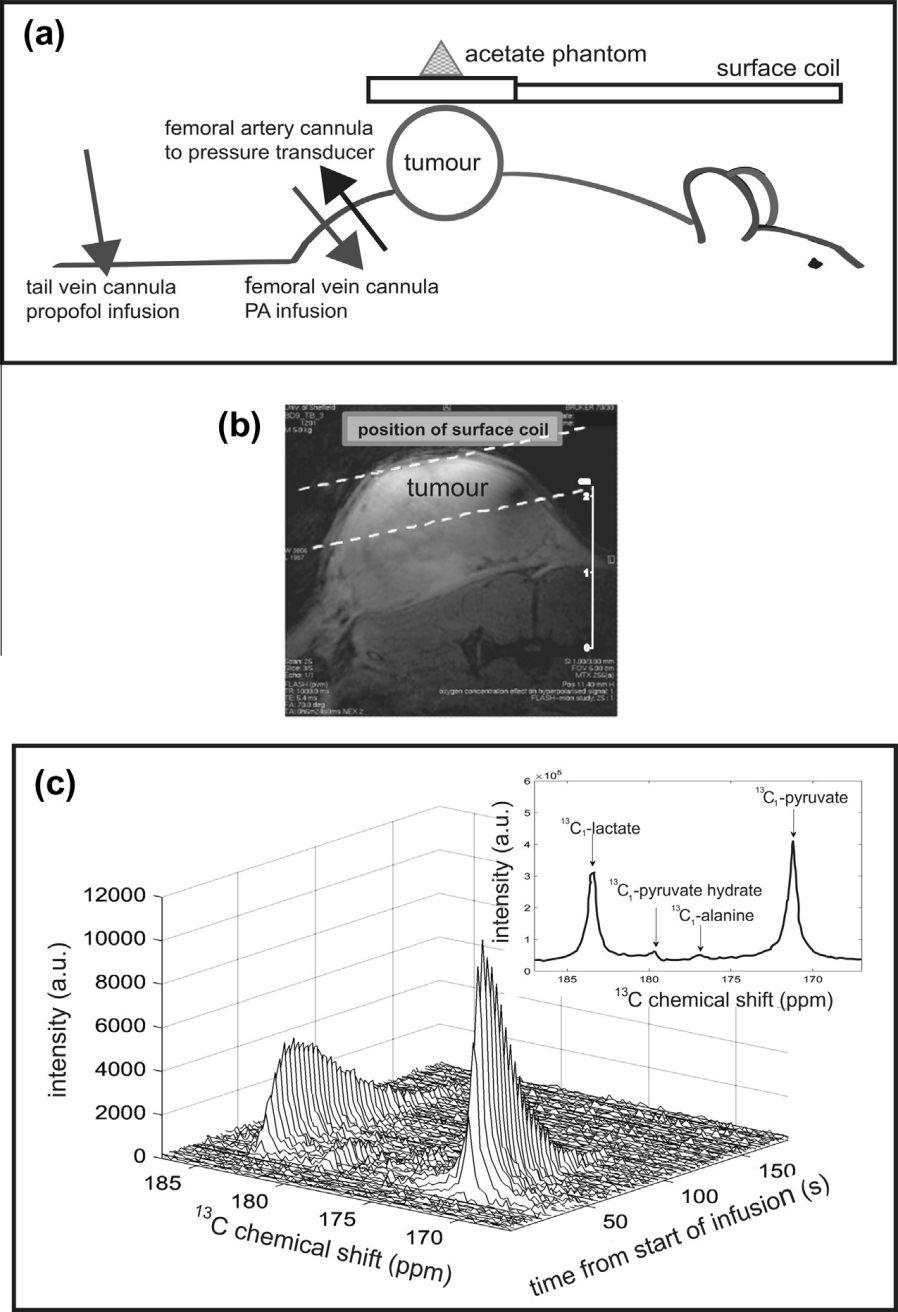
(a) Configuration of the animal setup in the magnet, showing location of surface coil, acetate phantom and cannulations, (b) axial FLASH image of the tumour. A cartoon representation of the surface coil location and guide-lines for the tumour slice are also shown. (c) Example of a series of acquired ^13^C spectra (absolute mode) versus time from the start of the infusion procedure (every third spectrum shown for clarity), from a rat under air-breathing conditions. Inset shows the ^13^C spectrum (absolute mode) as a sum of the time-course spectra.

A ^13^C/^1^H 20 mm surface coil (Bruker), positioned ∼1–2 mm above the tumour to minimise respiration artefact, was used for MR acquisitions (see Fig. 1a and b and Supplementary Materials for details). Briefly, a structural (FLASH) image was obtained to localise a slice for ^13^C-spectroscopy. ^13^C-MRS data acquisition commenced with automated transfer of PA from the polariser and infusion into the rat [18] via the femoral vein cannula (5 ml/kg, corresponding to 0.5–0.7 mmol/kg, over 13 s). Generally, this infusion rate had no effect on MABP. In the few cases where a minor increase was observed, return to base-line occurred within seconds. Each animal received two sequential PA infusions ∼60 min apart, both under air-breathing conditions in the normoxia group and under air-breathing followed by hypoxic conditions in the hypoxia group. Arterial blood was collected from the arterial cannula, starting at ∼3 min post PA infusion, and analysed for arterial blood oxygen tension (ArtpO_2_) using an ABL80 Flex blood gas analyser (Radiometer Ltd, Crawley, UK).

### MR data processing and kinetic analysis

MR raw data were processed using custom MATLAB software (MathsWorks Inc, Natick, MA). ^13^C_1_-pyruvate and ^13^C_1_-lactate peaks were integrated from phase-adjusted spectra for each acquired slice and time point (Fig. 1c). Signal integrals versus time were fitted to a previously validated precursor-product model using the pyruvate signal time course as an input function for lactate, from which the pyruvate to lactate conversion rate constant, *k_pl_*, was estimated [17]. Since pyruvate and lactate have a very similar molecular structure, it was assumed that changes in oxygenation (or any other microenvironmental factor) affect T1 and T2 of ^13^C_1_ labelled pyruvate and lactate equally.

### Biochemical analysis

Concentrations of pyruvate and lactate in P22 fibrosarcomas were measured in a control, untreated group and groups where the surgery, anaesthesia regime, timing of pyruvate infusions (not hyperpolarised) and gas breathing conditions used in the MR experiments were replicated. Rats were sacrificed ∼6 min after the final pyruvate infusion, where given. Tumours were rapidly excised, freeze-clamped in liquid nitrogen and stored at −80 °C before biochemical analysis. Pyruvate and lactate concentrations (performed at least in triplicate) were assayed spectrophotometrically, based on the levels of the co-factors, NAD^+^/NADH. See Supplemental Material for details.

### Statistical analysis

Analysis was carried out using MATLAB software. Paired or unpaired two-tailed Student’s *t*-tests, as appropriate, were used for testing the significance of differences between two groups. ANOVA with Bonferroni post hoc testing was used for multiple group comparisons. Linear regression analysis was used for correlations between physiological parameters – ArtpO_2_ and MABP. A *p* value of <0.05 was considered statistically significant.

## Results

### Tumour oxygenation and physiological status

**Supplementary Fig. S1:**
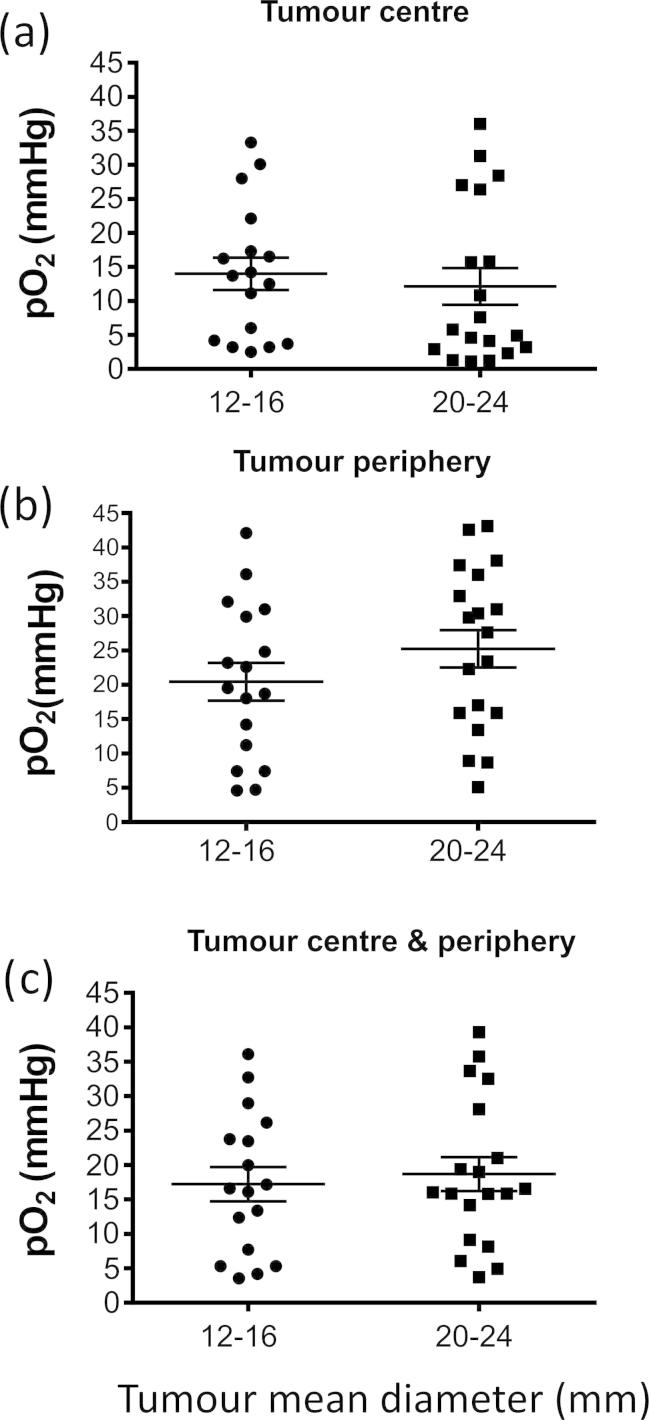


**Supplementary Fig. S2:**
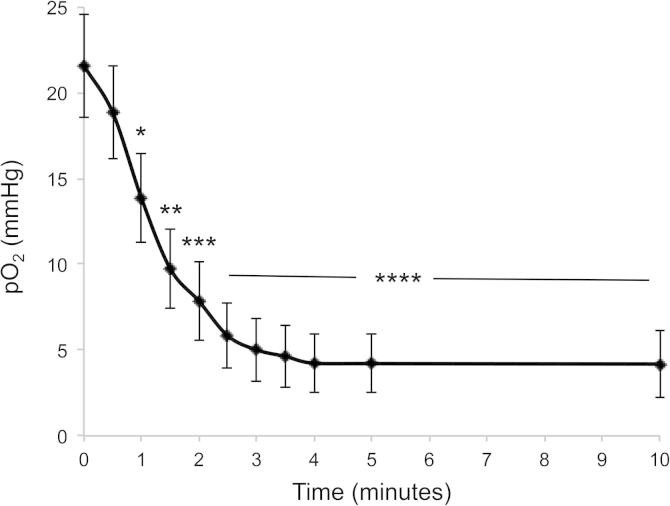


The basal local pO_2_ in P22 fibrosarcomas was 18.0 ± 1.7 mmHg (mean ± SEM), with no effect of tumour size in the range used (Fig. S1). Preliminary experiments established that ∼10% O_2_ in the inspired gas gave a rapid and reproducible average reduction in tumour pO_2_ of 82% without compromising the welfare of the animal. Tumour pO_2_ was significantly reduced at 1 min from the start of the hypoxic challenge (*p* < 0.05) and decreased further until response reached a plateau after 3 min (4.2 ± 1.7 mmHg; mean ± SEM; Fig. S2). 28% of the tumours had relatively hypoxic readings (∼5 mmHg) prior to the hypoxic challenge (Fig. S1c).

**Supplementary Fig. S3:**
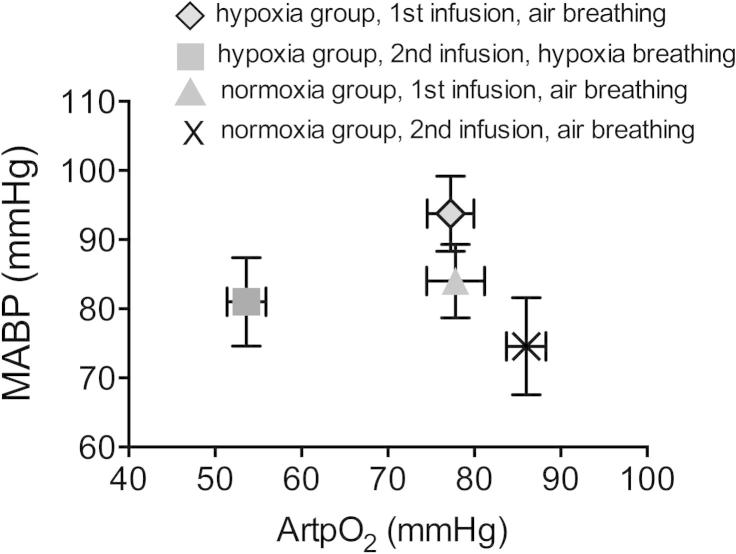


**Fig. 2:**
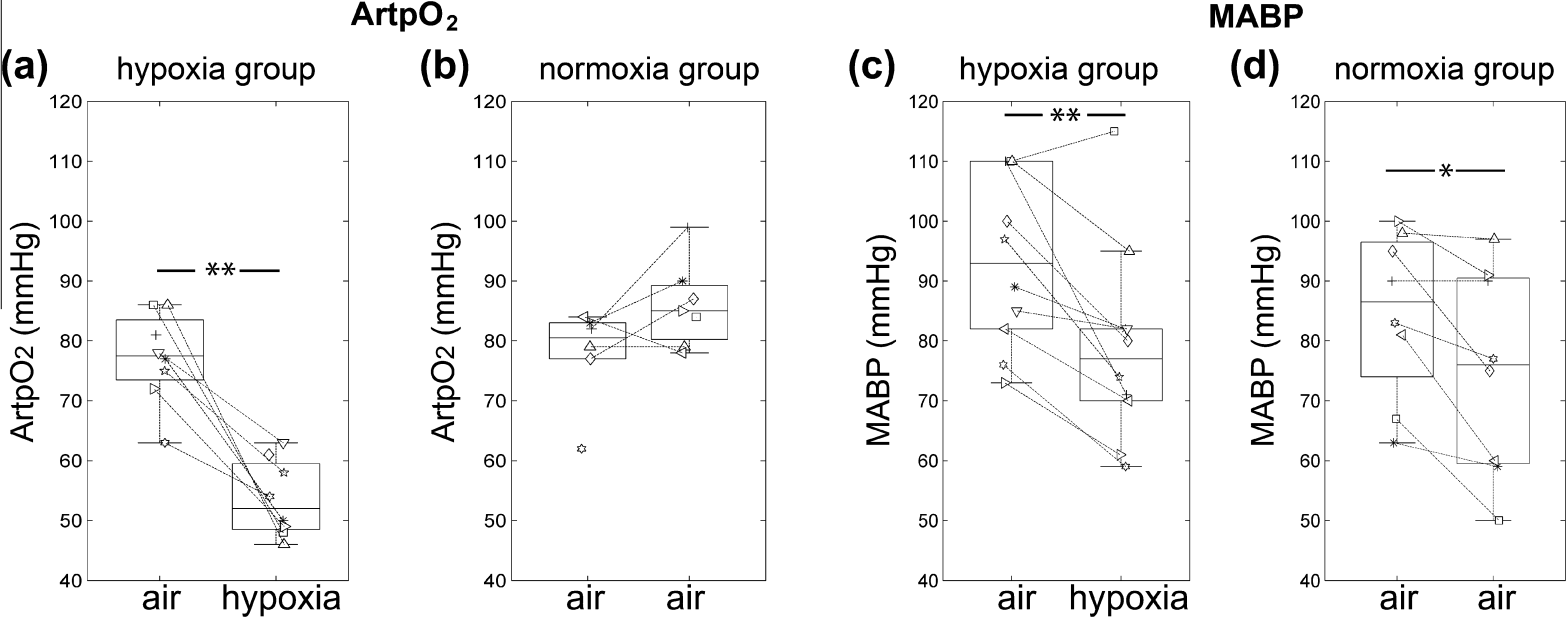
(a and b) ArtpO_2_ analysed after each PA infusion for the hypoxia group (*n* = 7 pairs) and normoxia group (*n* = 5 pairs). (c and d) MABP measured at the time of PA injection for the hypoxia group (*n* = 10 pairs) and normoxia group (*n* = 8 pairs). Each symbol represents an individual animal. Box plots show the median line, with the box edges representing the 25% and 75% quartiles. Whiskers extend to the furthermost value within 1.5 times the interquartile range from the 25% and 75% quartiles. Outliers are plotted beyond the whiskers. ^∗^*p* < 0.05; ^∗∗^*p* < 0.01.

ArtpO_2_ was significantly reduced in the hypoxia group following the 2nd PA infusion under hypoxia compared with that during the previous air-breathing phase (*p* < 0.01; Fig. 2a). In the normoxia group, infused twice under air-breathing conditions, there was no significant difference in ArtpO_2_ between the two measurements (Fig. 2b). Conversely, MABP was significantly decreased at the time of the second PA infusions, for both air-breathing and hypoxia-challenged animals (Fig. 2c and d). Although this effect tended to be larger under hypoxia, the decrease in MABP was not significantly different between the two animal groups and there was no direct correlation between MABP and ArtpO_2_ (Fig. S3).

### Hyperpolarised pyruvate experiments

**Fig. 3:**
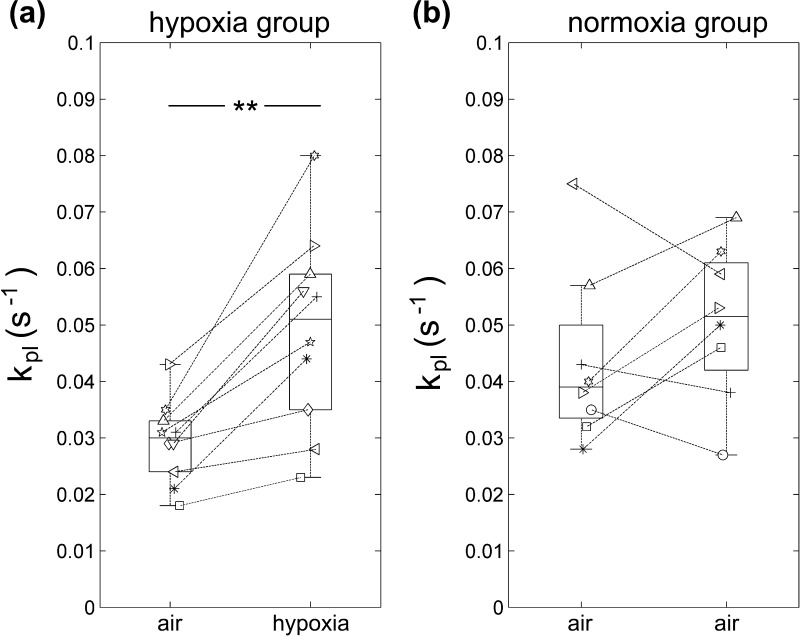
The rate constant for conversion of hyperpolarised ^13^C_1_-pyruvate to lactate, *k_pl_*, (s^−1^), in P22 fibrosarcomas for (a) hypoxia group (*n* = 10 pairs) and (b) normoxia group (*n* = 8 pairs). Each symbol represents an individual animal. Box plot representations of the data are described in Fig. 2. ^∗∗^*p* < 0.01.

The tumour pyruvate to lactate conversion rate constant, *k_pl_*, increased significantly (*p* < 0.01) from 0.029 ± 0.002 s^−1^ under air-breathing conditions (1st PA infusion) to 0.049 ± 0.006 s^−1^ under hypoxic conditions (2nd PA infusion) (mean ± SEM; Fig. 3a). There was also a tendency for *k_pl_* to increase between the two PA infusions in the normoxia group, where both infusions were administered under air-breathing conditions, but this was not statistically significant (*p* = 0.21; Fig. 3b). Unexpectedly, *k_pl_* for the 1st PA infusion was higher in the normoxia than in the hypoxia group (*p* < 0.05; Fig. 3), despite nominally the same air-breathing conditions.

### Relationship between *k_pl_* and physiological parameters

**Supplementary Fig. S4:**
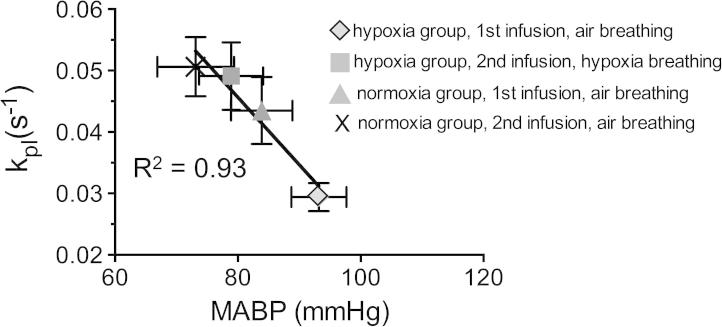


**Fig. 4:**
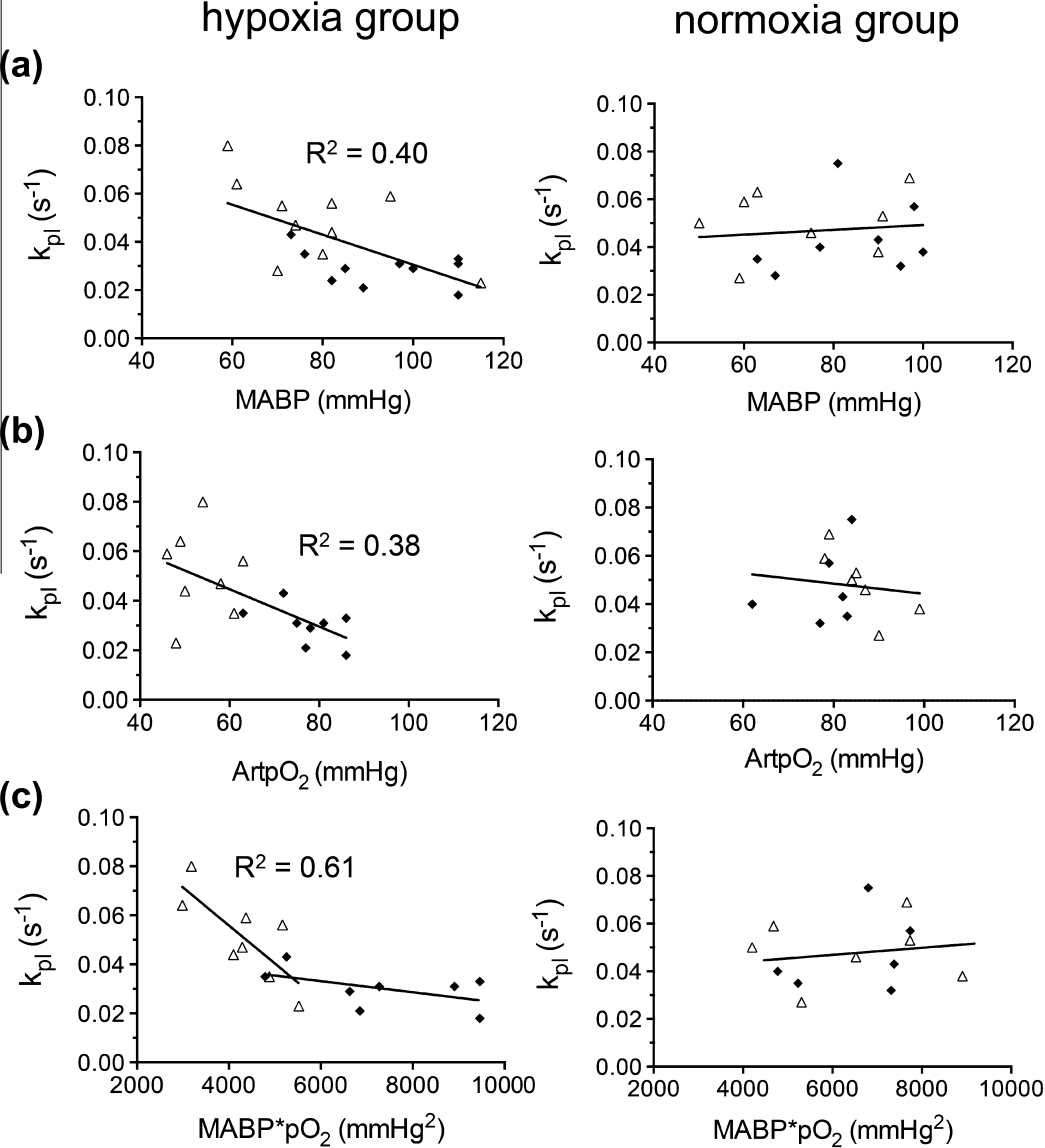
*k_pl_* versus (a) MABP; (b) ArtpO_2_ and (c) MABP * ArtpO2 for the hypoxia and normoxia groups. Each symbol represents an individual animal. Data are fitted to a linear model. Filled diamonds represent the 1st PA infusion, open triangles represent the 2nd PA infusion. *R*^2^ values are shown, where the slopes of the fitted lines are statistically different from zero (*p* < 0.05).

MABP and ArtpO_2_ were negatively correlated with *k_pl_* in the hypoxia group (*R*^2^ = 0.40; *p* = 0.003 and *R*^2^ = 0.38; *p* = 0.011 respectively) but not in the normoxia group (Fig. 4a and b). There was also a significant correlation between MABP and *k_pl_*, when the mean data from each of the acquisition groups were combined (*R*^2^ = 0.93; *p* = 0.035; Fig. S4). The product of MABP and arterial pO_2_ (MABP * ArtpO_2_) negatively correlated with *k_pl_* in the hypoxia group during the hypoxic challenge, where MABP * ArtpO_2_ values were below approximately 5000 mmHg^2^ (*R*^2^ = 0.61; *p* = 0.023) (Fig. 4c). No correlation was found between MABP * ArtpO_2_ and *k_pl_* in the normoxia group, consistent with the fact that MABP * ArtpO_2_ was generally greater than 5000 mmHg^2^ (Fig. 4c).

### Biochemical analysis

**Fig. 5:**
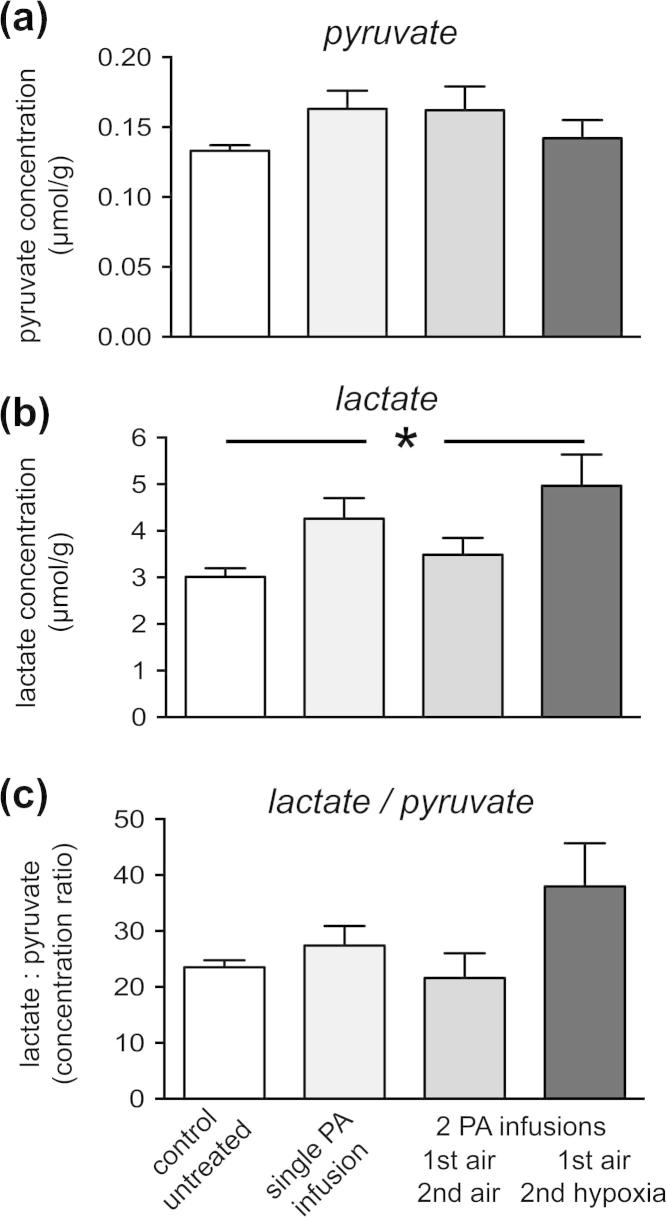
(a) pyruvate, (b) lactate concentrations in P22 fibrosarcomas determined by enzymatic assay in tumour extracts and (c) L/P ratio. Columns from left to right: the control group underwent no surgery, pyruvate infusions or gas challenges; rats received a single pyruvate infusion under air-breathing conditions (ArtpO2 = 87 ± 7 mmHg); rats received two sequential pyruvate infusions under air breathing conditions (ArtpO2 = 82 ± 10 and 87 ± 6 mmHg); rats received two pyruvate infusions, the first under air-breathing conditions, ArtpO2 = 87 ± 12 mmHg), the second breathing ∼10% O_2_, 4% CO_2_: balance N_2_ (ArtpO2 = 52 ± 11 mmHg). Data = mean ± SEM; *n* = 4–8 per group; ^∗^*p* < 0.05.

Results from freeze-clamped tumour tissue (16 ± 4 s from first incision to freezing; mean ± SD) showed that administration of exogenous pyruvate did not significantly increase tumoural pyruvate concentrations, though the variance between individual values within each group was increased (Fig. 5a). Lactate concentration was significantly higher in tumours during the hypoxic challenge, compared to tumours in untreated rats (4.97 ± 0.67 versus 3.01 ± 0.19 μmol/g wet weight; mean ± SEM; *p* < 0.05; Fig. 5b). There was also a tendency for lactate concentration and lactate:pyruvate ratio (L/P) to be higher in tumours in the hypoxia group, compared with the corresponding values in air-breathing groups, where rats received pyruvate infusions (Fig. 5b and c). However, these trends were not statistically significant.

## Discussion

We have detected a significant hypoxia-induced increase in the fractional rate constant (*k_pl_*) for conversion of pyruvate to lactate in tumour tissue, using hyperpolarised pyruvate (PA) and ^13^C MRS. We emphasise that *k_pl_* is a fractional rate constant (s^−1^) and that the rate of conversion of pyruvate to lactate (μmol s^−1^ (g tissue)^−1^) is given by the product of *k_pl_* and the tissue pyruvate concentration (μmol (g tissue)^−1^). No significant changes in the concentration of pyruvate were observed in any of the treatment groups after the administration of exogenous pyruvate, suggesting rapid basal turnover of pyruvate. The increase in *k_pl_* observed under hypoxic conditions therefore indicates an increase in the rate of conversion of pyruvate to lactate.

**Supplementary Fig. S5:**
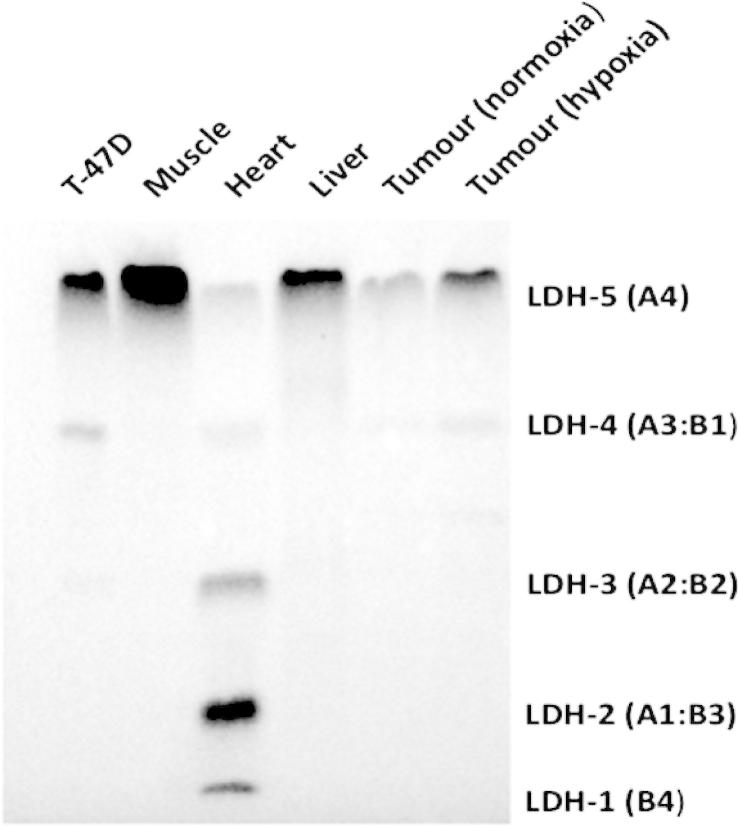


Allosteric inhibition of phosphofructokinase by ATP and citrate is the primary mechanism for the Pasteur effect. The inverse of this effect under hypoxia results in increased pyruvate production via glycolysis and its subsequent reduction to lactate, thus regenerating the NAD^+^ needed for glycolysis to proceed. Many other mechanisms, such as inactivation of the mitochondrial enzyme pyruvate dehydrogenase by hypoxia-induced reactive oxygen species [19] are also likely to impact on an increased lactate dehydrogenase (LDH)-catalysed lactate production under hypoxia. The ability of P22 tumours to respond to hypoxia by increasing the rate of conversion of pyruvate to lactate is also consistent with a predominant expression of LDH-5 (see Supplementary data, Fig. S5). All LDH isoenzymes have the ability to transform pyruvate to lactate and back [20] but the predominance of LDH-5 would favour the forward reaction. Our results are consistent with other studies, where tumour cell metabolism during hypoxia was investigated using MR-based methodology [14,21]. However, whilst long exposure to hypoxia can result in gene expression changes and up-regulation of proteins, the much shorter timescale of our hypoxic challenge can only be explained by a direct effect of hypoxia on pyruvate metabolism.

### Relationship between *k_pl_* and tumour lactate and pyruvate concentrations

Despite the increase in *k_pl_* observed under hypoxia, biochemical analysis of freeze-clamped tumours only showed a significantly higher concentration of tumour lactate in hypoxic animals compared to air-breathing animals, when the latter received no exogenous pyruvate (control untreated group, Fig. 5). Possible explanations are that excess pyruvate increased the rate of the LDH reaction towards lactate production or that the length of time under anaesthesia for pyruvate infusion induced hypoxia, even in the air-breathing animals. However, these factors would also apply in the MR experiments, suggesting that changes in *k_pl_* are a more sensitive index of induced hypoxia than changes in lactate concentration or the L/P ratio, even though pyruvate and lactate undergo rapid inter-conversion [22,23]. Consistent with our biochemical data, previous studies have shown that lactate concentration in tumours is dependent upon a complex interplay of factors, not just hypoxia [24–28]. For instance, excess lactate produced under hypoxia can be rapidly metabolised by well-perfused cancer cells, as a fuel source [29,30]. Alternatively, high levels of lactate can be rapidly exported into the blood supply for metabolism elsewhere, although we have not observed this in our previous experiments [17].

A significant increase in tumour *k_pl_* alongside only borderline increases in lactate concentration under hypoxia could also be explained by ^13^C spin exchange [31]. Here, ^13^C-lactate, metabolised from PA, enters the tumour lactate pool, where its concentration is much lower than the endogenous ^12^C-lactate concentration. Therefore, there is a higher probability that ^12^C-lactate, rather than ^13^C-lactate, is subsequently metabolised or exported, retaining ^13^C spins within the lactate pool. Temporarily higher lactate levels in tumours, under hypoxic conditions, would increase the likelihood of ^13^C label being retained within the large lactate pool, resulting in high estimations of *k_pl_*. In order to fully define the ultimate fates of pyruvate in tumours, simultaneous steady-state concentrations of metabolites and quantitative fluxes between different metabolic steps would need to be measured, as recently described for cancer cells in culture [32].

### Relationship between *k_pl_* and physiological parameters

The tendency for *k_pl_* to be higher for the second PA infusion in the normoxia group was most likely related to the measured decrease in MABP between the first and second acquisition; an expected effect of general anaesthesia (Fig. 2). Exogenously administered pyruvate, at a higher concentration than in our experiments, was also found to increase tumour hypoxia, albeit at later times than assessed in our experiments [33]. Overall, the data shown in Figs. 4 and Fig. S4 suggest that MABP does influence *k_pl_*. This is to be expected since MABP reflects tumour perfusion pressure and we previously showed that MABP correlates directly with blood flow in the P22 tumour [34]. Reduced tumour blood flow would, in turn, reduce oxygen delivery to the tumour, and therefore local pO_2_. In the hypoxia group, this effect would be exacerbated by the reduction in oxygen delivery caused by the decrease in ArtpO_2_. Indeed, the product of MABP and ArtpO_2_ was strongly influential on *k_pl_* in the hypoxia group, where ArtPO_2_ values dropped significantly. Unsurprisingly, this was not the case in the normoxia group, where ArtpO_2_ values were at physiological levels. General anaesthesia could have affected our results in other ways. For instance, propofol has been found to decrease mitochondrial function in certain cell/tissue types [35], although this would have been the case in both our treatment groups.

We found no correlation between ArtpO_2_ and MABP at the time of PA infusion (Fig. S3), although we did note a very rapid reduction in MABP immediately on switching to the hypoxic gas mixture (data not shown), consistent with compensatory vasodilation. The lack of correlation at later times is probably a reflection of the highly complex homoeostatic mechanisms controlling oxygen delivery to critical tissues and systemic blood pressure.

**Supplementary Fig. S6:**
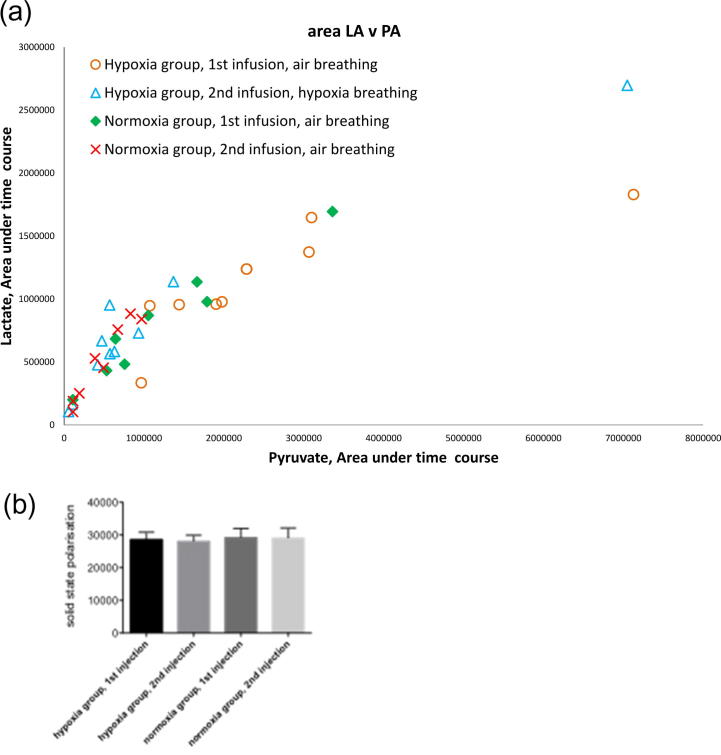


There was considerable inter-tumour heterogeneity in both tumour *k_pl_* (Fig. 3) and pO_2_ (Fig. S1) but whether there is a direct relationship between these two parameters is currently unknown. In any case, *k_pl_* for the 1st PA infusion in the hypoxia group was lower than *k_pl_* for the equivalent acquisition in the normoxia group, under the same nominal air-breathing conditions (Fig. 3). This discrepancy is likely to relate to the higher MABP recorded for the former group (Fig. 2), which suggests a higher blood flow rate and oxygen delivery, as noted above. The data shown in Fig. S6 show that the pyruvate signal, indicative to some extent of pyruvate delivery to the tumour, was generally highest for the 1st PA infusion in the hypoxia group, supporting this theory. Since animals were randomly allocated to groups, we can only assume that the difference in the MABPs between the two groups occurred by chance. Our data highlight the importance of measuring systemic parameters such as MABP and ArtpO_2_ to monitor the physiological status of animals throughout the duration of *in vivo* dDNP experiments.

### Limitations and further developments

One potential limitation of the dDNP technique is that it requires infusion of supra-physiological levels of ^13^C-pyruvate [33,36]. The size of the tumour pyruvate signal was quite variable in our experiments (Fig. S6a), despite the solid-state hyperpolarisation levels being very similar (Fig. S6b). Other factors such as tumour size, blood flow rate and coil position would impact on the size of signal obtained, whereas only blood flow rate would affect the actual concentration of pyruvate in tumour tissue. There was a non-linear increase in the lactate signal with increased pyruvate signal (Fig. S6a), which did not plateau. Although not definitive, these data suggest that tumour tissue is not saturated with pyruvate by the high dose of pyruvate administered. Partial tissue saturation was observed by Janich and colleagues [37] in the rat liver, kidney and heart with lower doses of pyruvate (0.2–0.4 mmol/kg) than used in the current study. Lower blood flow rate and thus slower dose delivery to tumours may explain these differences. Testing the validity of the common assumption that tumour microenvironmental factors such as oxygenation and pH affect ^13^C T1s of pyruvate and lactate equally would be informative, as differences would affect the *k_pl_* values obtained. Furthermore, our current procedure provides only a single estimate of *k_pl_* per tumour. In order to probe the well-known heterogeneity of the tumour microenvironment, an imaging based method such as spectral-spatial EPI [38] to highlight spatial variations in oxygenation changes would be a useful development of the current methodology.

## Conclusion

We have shown that the rate constant for pyruvate to lactate conversion, *k_pl_*, responds significantly to a rapid reduction in tumour oxygenation. A full quantitative analysis of pyruvate kinetics, including the acquisition of arterial input functions, would also enable pyruvate clearance from blood to tissue to be estimated. This provides the opportunity to investigate tumour vascular function, in addition to oxygenation-related metabolism changes, from a single PA infusion. Further studies incorporating simultaneous measurements of tumour pO_2_ and *k_pl_* are warranted and necessary to determine whether there is any relationship between *absolute* tumour pO_2_ and *k_pl_.*

Since tumours are highly dependent on pyruvate metabolism to lactate for progression, measurement of hyperpolarised pyruvate metabolism in tumours by ^13^C MRS is starting to be assessed as a way to monitor treatment efficacy. Our results emphasise the need to take oxygenation changes into account when interpreting dDNP data from such studies.

## Conflict of interest

The authors have no conflicts of interest to declare.
