# Supplementary material for: Measurement of the acute metabolic response to hypoxia in rat tumours in vivo using magnetic resonance spectroscopy and hyperpolarised pyruvate
Source: Radiother Oncol. 2015 Sep;116(3):392–9. doi: 10.1016/j.radonc.2015.03.011 (PMC4612449; doi:10.1016/j.radonc.2015.03.011)
Supplement: Supplementary data 1 [file mmc1.docx]

**Bluff, Reynolds *et al***

**SUPPLEMENTAL MATERIALS**

**Tumour pO_2_ measurements**

Local pO_2_ measurements were made in sub-cutaneous P22 fibrosarcomas in BDIX rats breathing air or reduced oxygen in the inspired gas. Rats were allocated to two tumour size groups with a mean diameter of 12-16mm (14.2±1.4mm mean±SEM; n=17) and 20-24mm (22.0±1.3mm mean±SEM; n=19), representing ellipsoid tumour volume group sizes of 905-2145mm^3^ and 4189-7238mm^3^ respectively (volume = 4.π/3.(d1.d2.d3)/8). Rats were anaesthetised with propofol (Rapinovet®, Intervet/Schering-Plough Animal Health, Milton Keynes, UK) infused via the femoral vein cannula at a rate of ~40 mg/kg/h; two OxyLite probes (Oxford Optronix Ltd, Oxford, UK), each incorporating a platinum-based fluorophore at the tip, were inserted per tumour. One probe was positioned at the tumour centre and the other at the tumour periphery. Local pO_2_ was calculated continuously from fluorescence life-time measurements, using an OxyLite/OxyFlo 4000 system, incorporating manufacturer calibration and data acquisition software (Powerlab Chart 4.2, AD Instruments Ltd, Oxford, UK). Figure S1 shows that mean tumour pO_2_, under air-breathing conditions, was not significantly different between tumours in the two different size groups. However, significantly different pO_2_ values were measured in the tumour centre compared to the tumour periphery (Student`s paired t-test, p<0.0005). This variance of pO_2_ distribution within the tumour was found for both tumour size groups (tumours with mean diameters of 12-16mm (p=0.0003) and 20-24mm (p<0.0001), respectively); Figure S1. In Figure S2, the decrease in tumour pO_2_ caused by switching to ~10% O_2_ is shown, as described in the main text.

**Hyperpolarisation of ^13^C_1_- pyruvate**

^13^C_1_-pyruvic acid (CIL, Andover; MA or Sigma Aldrich, Gillingham, UK) was mixed with 15mM OX063 trityl radical (Oxford Instruments, Abingdon, UK) and 1.5mM DOTAREM (Guerbet, Roissy, France). 44-50mg pyruvate was hyperpolarised using a HyperSense DNP polariser (Oxford Instruments, Abingdon, UK) for approximately 1 hour, until it reached >90% of maximum polarisation. Using 3.4ml superheated buffer solution (containing 40mM trizma pre-set crystals [pH 7.6], 0.269mM disodium EDTA and 50mM NaCl, all sourced from Sigma Aldrich, Gillingham, UK), the hyperpolarised frozen sample was dissolved to a final concentration of ~150mM and then transferred to an automated infusion system connected to the femoral vein cannula, within six seconds (Reynolds *et al*., 2012) ready for rapid infusion into the rat. The infusion system also contained a predetermined aliquot of 2.0M sodium hydroxide solution required to neutralise the PA. The PA dose to the animal was 0.5-0.7mmol/kg in a volume of 5 ml/kg.

**MR acquisitions**

Structural images were acquired using a FLASH sequence, with 6x6cm field of view, 512x512 matrix size, 30° flip angle (FA), 6ms echo time (TE) and 164.71 or 190.05ms repetition time (TR); 13 slices and 1mm slice thickness. For spectroscopy of PA metabolism in the tumour, an electronic trigger started both MRS acquisition and infusion over a 13 second period to ensure a reproducible infusion regime. A 0.5ml heparinised saline flush was given 10 minutes post-infusion of PA. ^13^C MRS acquisition used 8mm slice selection in the plane of interest, 20° FA, 1 ms Gaussian pulse, 1000 ms TR, 1.762 ms TE, 50 ppm sweep width, 512 time domain points and 180 consecutive spectra. A 6.7M sodium ^13^C_1_-acetate phantom placed on the surface coil (see Figure 1 of the main text) was used as a reference signal for spectroscopy.

**Biochemical analysis**

Tumour fragments were weighed and ground to powder in liquid nitrogen before homogenisation in 7% perchloric acid (PCA), centrifugation and neutralisation with 2M KHCO_3_ (Sigma Aldrich, Gillingham, UK). Tumour extracts were stored at -80^o^C prior to biochemical analysis. Assays for pyruvate and lactate were performed spectrophotometrically, based on the levels of the NADH and NAD co-factors, respectively. For lactate, 20mg of NAD (Sigma Aldrich, Dorset, UK) was combined with 8ml of deionised water and 4ml of 0.6M glycine/0.5M hydrazine buffer (pH 9.2) and 200µl bovine heart L-lactic dehydrogenase (LDH). 1.45ml of the NAD solution was mixed with 50µl of tumour PCA extract, incubated for 30 minutes at room temperature and the absorbance at 340nm (compared to no tumour control) was measured using a Cary60 spectrophotometer (Agilent Technologies, Edinburgh, UK). For pyruvate, 1mg of NADH was combined with 2.2ml 1.5M Trizma base solution (Sigma Aldrich, Gillingham UK). Tumour PCA extract volume was determined empirically, based on measurements on previous samples, to ensure the sensitivity limit of the assay was met. Equal volumes of 1.5M Trizma base solution and NADH dissolved in Trizma solution (250μl) were mixed with the PCA extract and the absorbance at 340nm was measured (compared to deionised water control). Following this, 25μl of LDH was added, agitated and absorbance at 340nm measured for 15 minutes. The change in absorbance observed was determined by the pyruvate concentration.

**Influence of arterial pO_2_ (ArtpO_2_) on mean arterial blood pressure (MABP)**

There was no clear relationship between ArtpO_2_ and MABP, when ArtpO_2_ was measured immediately after PA administration and MABP was measured during PA administration, as shown in Figure S3. See main text for Discussion.

**Influence of MABP on *k_pl_***

*k_pl_* correlated with MABP for individual tumours in the hypoxia group (Figure 4 in the main text) and for the mean values of the different groups (Figure S4). See main text for Discussion.

**Zymography for lactate dehydrogenase isoenzymes**

Zymography to determine the LDH isoenzymes present in our tumour model was performed using cellulose acetate membranes (Titan III 94x76mm; Helena Biosciences, Tyne and Wear, UK) with Tris-Glycine buffer, according to the supplied manufacturer’s instructions and as previously described (Glen *et al*., 2008). Following electrophoresis, the gels were placed in staining solution (5mM NAD, 50mM lithium lactate, 0.1mM Tris-HCl (pH 8.6), 0.2mM phenazinmethosulphate (PMS), 2mM nitrobluetetrazolium (NBT) and 0.8mM (3-(4,5-dimethylthiazol-2-yl)-2,5-diphenyltetrazolium bromide (MMT) and the colour allowed to develop in the dark at 37°C (~3-6 minutes). See Figure S5.

**Pyruvate versus lactate dose response**

Figure S6a) shows the area under the lactate MR time-course versus area under the pyruvate MR time-course, in arbitrary units, for tumours in animals in the normoxia and hypoxia groups. There was a non-linear increase in lactate area, as pyruvate area is increased, with no plateau (Figure S6a). Factors that affect pyruvate area include variation in polarisation levels, coil position, tumour size, tumour blood flow and tissue heterogeneity. However, solid state polarisation was very similar for all groups, with little variation between individual scans within a group (Figure S6b), suggesting that polarisation in the liquid phase was similarly uniform – see main text for Discussion.

**Supplemental figure legends**

Figure S1: pO_2_ recordings in P22 fibrosarcomas of different sizes, with the OxyLite system (n=36) from 1 probe positioned at the tumour centre (a) and 1 probe positioned at the tumour periphery (b). Averaged data from tumour centre and periphery are shown in c). No significant effect of tumour size was observed. Data show the individual values and mean± SEM.

Figure S2: pO_2_ recordings in P22 fibrosarcomas (mean±SEM) in response to 10% O_2_ breathing (n=18). Tumour pO_2_ was significantly reduced over time. *p<0.05; **p<0.01; ***p<0.001; ****p<0.001.

Figure S3: Relationship between MABP and ArtpO_2_. Means±SEM are shown for the different treatment groups; n=6-8 per group. No significant correlation was observed.

Figure S4: Relationship between k_pl_ and MABP. Means±SEM are shown for the different groups; n=8-10 per group. A significant correlation was observed.

Figure S5: Zymography for LDH isoenzymes in rodent tissue. The expression of all 5 LDH isoenzymes can be seen in BDIX rat heart. In contrast, a human breast cancer cell line (T-47D), BDIX rat muscle, liver or P22 sarcomas express predominantly LDH-5 (LDH-A4), with absent or relatively lower levels of LDH-4 (LDH-A3:B1) under both air-breathing (tumour normoxia) and hypoxic gas breathing conditions (tumour hypoxia; ~10% O_2_; 4% CO_2_: balance N_2_).

Figure S6: a) Pyruvate versus lactate area under time-course curve for all animals in the MR experiments. Each point represents an individual animal. b) Solid state polarisation levels for each treatment group; means±SEM for each treatment group; n=8-10 per group.

**References**

Glen A, Gan CS, Hamdy FC et al. iTRAQ-facilitated proteomic analysis of human prostate cancer cells identifies proteins associated with progression. J Proteome Res 2008;7:897-907.

Reynolds, S., Kazan, S., Bluff J et al. Fully MR compatible syringe pump for the controllable injection of hyperpolarized substrate in animals. Appl Magnetic Resonance 2012;43:263-273.
